# Incidence, Pathotyping, and Antibiotic Susceptibility of Avian Pathogenic *Escherichia coli* among Diseased Broiler Chicks

**DOI:** 10.3390/pathogens9020114

**Published:** 2020-02-12

**Authors:** Ashraf M. Awad, Nahed A. El-Shall, Doha S. Khalil, Mohamed E. Abd El-Hack, Ayman A. Swelum, Ahmed H. Mahmoud, Hossam Ebaid, Ahmed Komany, Reda H. Sammour, Mahmoud E. Sedeik

**Affiliations:** 1 Department of poultry and fish diseases, Faculty of Veterinary Medicine, Alexandria University, Edfina, Elbehira 22758, Egypt; 2 Department of Poultry, Faculty of Agriculture, Zagazig University, Zagazig 44511, Egypt; 3 Department of Theriogenology, Faculty of Veterinary Medicine, Zagazig University, Zagazig 44511, Egypt; 4 Department of Zoology, College of Sciences, King Saud University, Riyadh 11451, Saudi Arabia; 5 Department of Botany and Microbiology, College of Science, King Saud University, Riyadh 11451, Saudi Arabia

**Keywords:** *E. coli*, *APEC*, serotyping, PCR, virulence gene, antibiotics, broilers, resistance

## Abstract

A total of 54 broiler flocks during the first two weeks of life was used to investigate the incidence of avian pathogenic *E. coli* in Egypt; 28 isolates (51.85%) were revealed by colony morphology and biochemical identification which then investigated for their serogroups and only 18/28 isolates were serotyped. The most prevalent serotypes were O115, O142, O158, O55, O125, O114, O27, O20, and O15. By application of polymerase chain reaction (PCR), 83.3% (15/18) of the serotyped isolates were confirmed to be *E. coli*, and 93.3% (14/15), 46.6% (7/15), and 20% (3/15) of isolates harbored the *iss*, *iutA*, and *fimH* genes, respectively. Virulence testing of the selected 13 *APEC* isolates on the specific-pathogen-free (SPF) chicks revealed them to be highly virulent (15.4%), moderately virulent (23.1%), and avirulent (61.5%); however, all isolates (100%) were extremely virulent towards SPF embryonated chicken eggs. Antibiotic resistance (100% of isolates (n = 13)) was observed for ampicillin, amoxycillin–clavulanic acid, and tetracyclines, colistin (92.31%; 12/13), doxycycline and spiramycin (84.62%; 11/13), florfenicol (69.23%; 9/13), cefotaxime (61.54%; 8/13), and ciprofloxacin (53.85%; 7/13). The highest percentage of sensitivity (53.85% of isolates; 7/13) was recorded for ofloxacin and enrofloxacin followed by gentamycin (46.15%; 6/13). The results suggest that the diagnosis of *APEC* with PCR is rapid and more accurate than traditional methods for *E. coli* identification; moreover, the presence or absence of *iss, iutA,* and/or *fimH* genes is not an indicator of in vivo pathogenicity of *APEC*. Thus, further studies, including a wider range of virulence genes and gene sequencing, are required. In addition, serotyping has no effect on the virulence of *APEC*.

## 1. Introduction

*Escherichia coli (E. coli)* is a Gram-negative bacterium of the family *Enterobacteriaceae* [1]. It is a commensal microorganism found in the intestine of humans and animals; however, it may induce illness so they are classified to commensal and pathogenic *E. coli* with further classification of pathogenic group to two pathotypes, diarrheagenic *E. coli* (DEC) and extraintestinal pathogenic *E. coli* (ExPEC), that cause various diseases in both humans and animals [2]. Avian colibacillosis, caused by ExPEC, is one of the major bacterial diseases in the poultry industry that has gained immense attention worldwide [3]. It is responsible for various disorders, including colisepticemia, air sacculitis, peritonitis, perihepatitis, pericarditis, omphalitis, coligranuloma, enteritis, synovitis, swollen head syndrome, and osteomyelitis [4], which eventually lead to total or partial condemnation of carcasses and expensive antibiotic treatment [3]. Animals of various ages are susceptible to avian colibacillosis; adults are more prone to infection. Moreover, yolk sac infection and high mortality rate in the baby chicks or embryos were recorded following the penetration of eggshells with *E. coli*, as well as spreading of the organism during egg hatching, laying, or oviduct formation [3,5,6]. Furthermore, it is important to consider the potential for its zoonotic transmission through poultry reservoirs [7]. Hence, the European Union has enforced food safety legislation, which usually constitutes a blueprint for the bill in third countries [8,9]. For determining the virulence of *E. coli* strains, its inoculation into embryos or 1-day-old chicks is followed as a golden standard test [10], whereas serotyping remains the most frequently used diagnostic method in laboratories, although it only allows the identification of a limited number of avian pathogenic *E. coli (APEC*) strains [11]. Moreover, the prevalence of a certain serotype is linked with the geographical localization of a flock [3]; hence, various molecular typing methods have been employed to study *APEC*, but none has revealed a specific genotype [12]. Several virulence genes have been associated with *APEC* including adhesin factors such as (*fimH*) that relate to the adhesion to the avian upper respiratory tract [13], as well as *iss* gene, which is known for its increasing ability to survive in the serum and it has been found more frequently among the pathogenic strains than the nonpathogenic ones, [14]. The *iutA* gene, one of the five genes of the aerobactin operon, encodes an outer membrane protein involved in the high binding affinity for iron [15,16]. Previously, the frequency of these genes was higher in *APEC* isolates obtained from other countries. Number and combination patterns of the virulence-associated genes in *E. coli* strains correlate with the virulence factors [17]; nevertheless, numerous studies have demonstrated that these virulence factors are rarely present in the same isolate and they can occur either individually or polygenically with varying frequencies in the clinical isolates [18,19]. This study aims to investigate the prevalence of *APEC* in baby chicks using traditional laboratory methods, followed by confirmatory molecular techniques, and assess their virulence in specific pathogen-free (SPF) 1-day-old chicks and embryonated chicken eggs (ECEs) and the correlation of in vivo virulence with studied virulence genes (*iss*, *iutA*, and *fimH*) or serotypes. Finally, the isolates are subjected to antibiotic sensitivity testing.

## 2. Results

### 2.1. Bacterial Isolation, Identification, and Serogrouping

The cultural morphology on MacConkey and Eosin Methylene Blue (EMB) agars and biochemical identification revealed that 51.85% (28/54) of the examined broiler chick flocks were positive to *E. coli.* Serological identification revealed that 18/28 isolates of *E. coli* were serotyped (64.3%), whereas 10/28 isolates were untypable (35.7%). The most prevalent serotype was O115 4/18 (22.2%); followed by O142 3/18 (16.66%); O158, O55, O125, and O114 2/18 (11.11% for each); and O27, O20, and O15 1/18 (5.55% for each).

### 2.2. Polymerase Chain Reaction (PCR) Using E. coli Species-Specific Gene and Virulence Genes (iss, iutA, and fimH)

**Figure 1:**
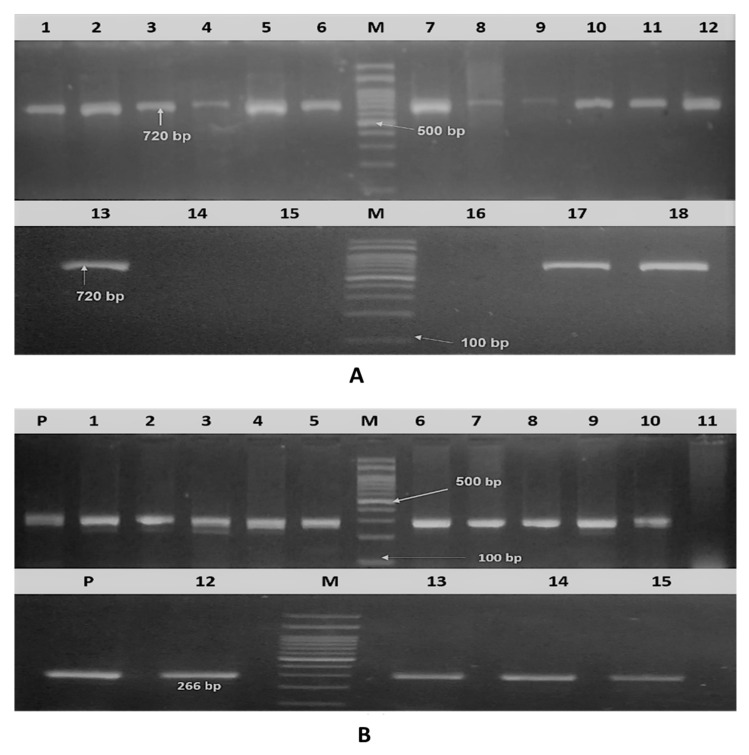
Agarose gel electrophoresis. (**A**) Amplified *PhoA* gene of isolated APEC (18 serotyped isolates). Lane M: DNA molecular weight ladder (100 bp ladder), lane 1–18: isolates and positive sample at 720 bp. (**B**) Amplified *iss* gene of 15 molecularly confirmed APEC. Lane M: DNA molecular weight ladder (100 bp ladder), lanes p: positive control (266 bp), lane 1–15: samples.

**Figure 2:**
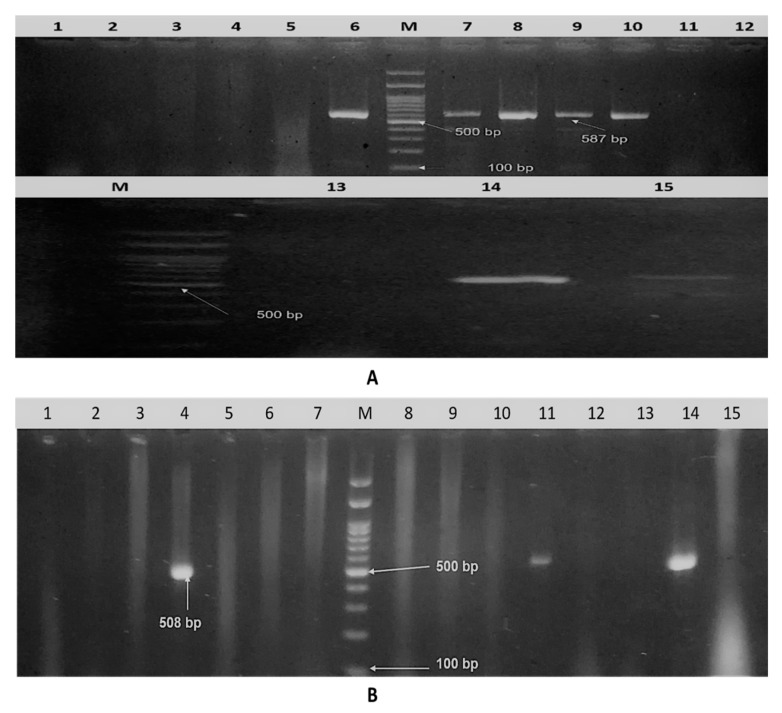
Agarose gel electrophoresis. (**A**) Amplified *iutA* gene of isolated APEC. Lane M: DNA molecular weight ladder (100 bp ladder), lanes 1–15: samples and positive samples at 587 bp. (**B**) Amplified *fimH* gene of isolated APEC. Lane M: DNA molecular weight ladder (100 bp ladder), lane 1–15: samples, and positive sample at 508 bp.

Of the 18 (83.3%) isolates, 15 were confirmed to be *E. coli* (positive for *phoA* gene; Figure 1A). Considering the virulence genes, the *iss* gene was detected in 14/15 isolates (93.3%), 7/15 (46.6%) were positive for the *iutA* gene, and 3/15 (20%) were positive for the *fimH* gene, as presented in (Figure 1B and Figure 2A,B). Various gene combinations were observed as following: *iss* gene was alone present in 6/15 of isolates (40%), *iutA* in 1/15 isolates (6.7%), *iss plus fimH* in 2/15 (13.3%), *iss* plus *iutA* in 5/15 (33.3%), and the three virulence genes were detected in 1/15 isolates (6.7%). 

### 2.3. Results of Virulence Assessment in Specific Pathogen-Free (SPF) 1-day-old Chicks and Embryonated Chicken Eggs (SPF ECEs)

In vivo virulence assessment of thirteen *E. coli* isolates that represent different combinations of serotypes and virulence genes was performed (Table 1). Five isolates recorded the mortality rate as 20%, 40%, 40%, 60%, and 100%, respectively, with lesions including pericarditis, perihepatitis, air sacculitis, liver necrosis, and pneumonia, whereas the other 8 isolates recorded no mortalities but postmortem lesions included mild air sacculitis at the 7th day post-inoculation (PI) of SPF 1-day old chicks. In SPF ECEs, all 13 isolates resulted in (40–100%) embryo mortality, with the highest mortality rate at day 2 PI; cranial and skin hemorrhages and liver necrosis were recorded.

### 2.4. Results of Antibiotic Sensitivity Testing

Susceptibility of 13 *E. coli* isolates to 13 antibiotics was recorded and revealed that 7/13 (53.84%) of isolates were sensitive to ofloxacin and enrofloxacin, followed by gentamicin (6/13; 46.15%), ciprofloxacin and florfenicol (3/13; 23.08%), doxycycline, neomycin, and cefotaxime (1/13; 7.69%). All isolates recorded 100% resistance to ampicillin, amoxycillin–clavulinic acid, and tetracycline, followed by colistin (92.31%), spiramycin and doxycycline (84.62%), and florfenicol (69.23%); moreover, the intermediate susceptibility was showed by 53.85% of isolates for neomycin, 30.77% for cefotaxime, 23.08% for ciprofloxacin, and 15.38% for ofloxacin. Two isolates (code no. 50 and 7) showed resistance to all tested antibiotics, as listed in Table 2.

## 3. Discussion

In this study, bacteriological investigation for *APEC* was performed on 54 broiler flocks in the northern delta of Egypt during the first two weeks of age to determine the most prevalent serotype and virulence genes associated to these serotypes; furthermore, in vivo assessment of the virulence of certain selected isolates and their in vitro antibiotic sensitivity testing was carried out. 

Although all examined diseased field broiler flocks (n = 54) received antibiotics during the last three days just before sampling, 51.85% of these flocks were positive to *APEC* by culturing and biochemical identification; it may indicate antibiotic resistance in the field. On the other hand, negative flocks (48.45%) may exhibit *APEC* but it is possible to suppress using antibiotics; unfortunately, this is what happens in the field before coming into clinical laboratories. The most common isolated serotype was O115 (14.2%); followed by O142 (10.7%); O158, O55, O125, and O114 (7.1% for each); and O27_,_ O20_,_ and O15 (3.5% for each); whereas, O1, O2, and O78 serotypes represent 0%. Nevertheless, similar findings regarding serotype prevalence were recorded by Abdeltawab et al. [24], Abd El-Haleem et al. [25], and Roshdy et al. [26]; however, the geographical localization of a flock may affect the prevalence of certain serotype. The observable decreased rate of O78 incidence may be attributed to the use of vaccines containing this serotype [11]. 

The observed percentage of the untypable isolates (35.7%) by O sero-grouping may be the result from autoagglutination or an incomplete antisera panel [27]; this was in accordance with the study by Eid et al. [28] and Abd El-Haleem et al. [25]. Eighteen *APEC* isolates were identified by serotyping; nevertheless, the use of the PCR technique revealed that only 15/18 (83.3%) isolates were positive for *phoA* gene (species-specific gene), and this method was repeated thrice for best results. These results indicate that molecular identification is more accurate and faster than the traditional method of bacteriological identification (colony morphology, biochemical, and serotyping); Eid et al. [28] reported similar findings. 

Holland et al. [29] and Chui et al. [30] mentioned that the detection of specific microorganisms depends on virulence genes, and neglecting the species-specific gene is not recommended due to the presence of microorganisms that have similar virulence traits rather than the target microorganism itself. So in this study, the detection of the species-specific gene is considered the main step to complete the investigation of virulence genes. The *iss*, *fimH*, and *iutA* genes were selected due to their common prevalence in *APEC* isolated from broiler samples via PCR [19,31,32,33]. Our findings revealed that the most prevalent virulence gene was *iss* (93.3%), followed by *iutA* (46.6%), and *fimH* gene (20%); this highest incidence of *iss* and *iutA* genes among the pathogenic cases was in accordance with that in the study by Rodriguez-Siek et al. [34] (81.5% and 80.2%, respectively) and Rocha et al. [35] (73.8% and 45.9%, respectively). In contrast, Delicato et al. [19] detected the *iutA* gene with a higher percentage (63%) than the *iss* gene (38.5%). 

The adhesion gene (*fimH*) showed low prevalence (20%), which was in accordance with the result (33.3%) reported by Mbanga and Nyararai [33]; though it was 98.1% [35] and 96.5% [19], these varying proportions were observed as this gene is not the sole adhesion factor. Several other genes such as P fimbrial adhesins, F11, Curli fimbriae, and other adhesins [36] possess adhesion properties; these are commonly found in *APEC* isolated from septicemic cases [37], and play a pivotal role in stabilizing *APEC* infection. 

Several studies compared different models of pathogenicity testing, for example, the study of Gibbs et al. [38], which stated that intravenous, subcutaneous, and intratracheal challenges of chickens were strongly correlated with the embryo lethality assay. Moreover, the latter can discriminate between highly virulent, moderately virulent, and avirulent isolates of avian *E. coli* by determining the morality percentage [13]. 

Virulence assessment of *APEC* isolates in 1-day-old chicks or embryos is considered as the gold standard test to detect virulent strains according to the ability of *APEC* isolates to kill chicks or embryos in each model [39]. The virulence of 13 *APEC* isolates that were selected to be representative of different serotypes and virulence gene content in this study was compared using these two models.

In subcutaneously inoculated SPF 1-day-old chicks, two isolates were classified as highly virulent, three isolates were moderately virulent, and the remaining eight isolates were avirulent and showed mild air sacculitis without mortalities. In contrast, all the 13 tested isolates were highly virulent following the allantoic sac inoculation of SPF ECEs. This variation in mortality and the virulence between the embryo and chick models may indicate that the survival frequency of chicken embryo against colibacillosis lesions is less than that of chicks [40], which may be a result of the highly developed immune system of chicks than that of the embryo.

All 13 isolates, with variable serotypes, were highly virulent according to the SPF ECEs model; furthermore, in 1-day-old chicks, different isolates of the same serotype revealed distinct virulence indices; for example, two isolates of each of O114 and O158 were low and moderately virulent, thus indicating that serotyping has no effect on the virulence of *APEC*. Previous reports [11,41] proved that serotyping did not distinguish between the pathogenic and nonpathogenic *E. coli*.

In addition, it was observed that some highly virulent isolates in the SPF 1-day-old chick model had only one virulence gene, whereas others with all three investigated genes did not result in mortality (low virulent). Hence, no correlation existed between the number of virulence genes and virulence that agreed with the findings of previous studies [11,19], which reported that the presence of a single or even a combination of virulence genes is not enough to investigate most of *APEC*. Moreover, there are other genes that may be integrated in virulence other than *iss*, *iutA,* and *fimH*, and a sequence variation of the same gene may also play a role. Moreover, Wang et al. [17] suggested the presence of a real interaction among the *APEC* virulence factors; however, this role has not yet been established. Therefore, further studies are warranted to investigate these points.

In poultry production, the primary step for controlling losses resulting from *APEC* infection is to use antimicrobial therapy; however, the antimicrobial resistance increased with time among several bacterial species and resulted in major health threats [42]. Studying the susceptibility of 13 *APEC* isolates for 13 antibiotics revealed that the organism was susceptible to ofloxacin and enrofloxacin (53.85%), followed by gentamicin (46.15%), ciprofloxacin and florfenicol (23.08%), and eventually, neomycin, cefotaxime, and doxycycline (7.69%); however, 92.31% of the isolates exhibited resistance for colistin and 84.62% for spiramycin and doxycycline. Interestingly, resistance to all tested antibiotics was recorded for two isolates and three isolates had sensitivity to only 1/13 of the antibiotics. Moreover, all isolates (100%) exhibited resistance to ampicillin, amoxycillin–clavulanic acid, and tetracyclines. This issue is of great concern and a high-risk worldwide as even mild bacterial infections cannot be treated with antibiotics. Sepehri and Abbass-Zadeh [43] reported that the highest resistance was recorded for oxytetracycline, flumequine, neomycin, difloxacin, and enrofloxacin. Moreover, Ozawa et al. [44] reported that more than 70% isolates were resistant to ampicillin and tetracycline; this is of importance as penicillins and tetracyclines, in addition to sulfonamides and streptomycin, are the oldest drugs used against infectious bacterial diseases. Hence, resistance to these drugs was expected [45,46], and uncontrolled use of antimicrobials in poultry production for disease treatment or prevention may result in drug resistance, which was reported in our study. The use of antimicrobials as medication should only be for infectious cases to prevent their misuse, and applying biosecurity measures to overcome infection and decreasing the need for these antibiotics are crucial measures for public health.

## 4. Materials and Methods

In this study, all experimental procedures were performed according to the Local Experimental Animal Care Committee and were approved by the ethics board of the Institutional Committee of the Department of Poultry and Fish Diseases, Faculty of Veterinary Medicine, Alexandria University, Egypt. All efforts were made to minimize the suffering of animals.

### 4.1. Sample Collection

Liver, heart blood, and spleen pooled samples were collected from 54 broiler chicken flocks during the first 2 weeks of age from the governorates (Alexandria and El-Behera) of North Delta, Egypt, under a complete aseptic condition in the laboratory of the Poultry and Fish Diseases Department, Faculty of Veterinary Medicine, Alexandria University. For bacteriological examination, these samples were collected from live, diseased cases (5–7 chicks/flock) by postmortem examination; they exhibited omphalitis, perihepatitis, pericarditis, and air sacculitis as listed in Table 3.

### 4.2. Bacteriological Isolation, Identification, and Serotyping

A loopful sample from each specimen was inoculated into the nutrient broth and was incubated at 37 °C for 24 h; thereafter, a loopful of inoculated broth was streaked on MacConkey agar plates and incubated for 24 h at 37 °C. Suspected lactose fermented colonies were isolated and streaked on eosin methylene blue media (EMB). Next, suspected colonies from EMB were isolated and subcultured into nutrient agar slants and were incubated at 37 °C for 24 h and then stored at 4 °C until use for further biochemical testing such as indole, methyl red, citrate utilization, urease, triple sugar iron (TSI), catalase, and Voges Proskauer tests.

In total, 28 isolates that were identified biochemically as *E. coli* were subjected to serological identification using slide agglutination test according to Lee et al. [47] using specific eight polyvalent, then 43 monovalent group O somatic antisera (Denka Seiken Co., Ltd. Tokyo, Japan), as shown in Table 4. The serotyping was performed in the serology unit, Animal Health Research Institute, Dokki, Egypt.

### 4.3. Polymerase Chain Reaction (PCR) Using E. coli General Gene and Virulence Genes (iss, iutA, and fimH)

Eighteen serotyped *APEC* isolates were tested for the presence of *phoA* gene, encoding *E. coli* species, by PCR technique. Moreover, virulence genes including *iss*, *iutA,* and *fimH* were detected on PCR-confirmed *APEC* isolates.

DNA extracts were prepared by the boiling method according to Sambrook et al. [48]. PCR reaction comprising 1 µL of extracted DNA was used with PCR Master Mix (10 µL; 2X TOP simple™ dyemix–ntaq cat.#p510t), forward primer (1 µL), reverse primer (1 µL ), and distilled water up to a volume of 20 µL. Primer sequence and cycling conditions are listed in Table 5 and Table 6.

### 4.4. Virulence Testing APEC Isolates

The virulence of 13 *APEC* isolates was assessed; these isolates were selected to be representatives according to the serotype and virulence genes content (isolate no. 9, 16, 41, 50, 28, 54, 5, 2, 7, 39, 15, 32, and 49), as listed in Table 1 by the following models:

- **One-day-old specific pathogen-free (SPF) chick model:** Seventy-five 1-day-old SPF chicks, supplied by a SPF farm at Al-fayoum province, Egypt, were divided into 15 groups (*n* = 5) for {13 isolates, 2 control groups (one sham-challenged subcutaneously with saline and the other non-challenged}. All chicks were reared in separate cages with food and water supplied ad-libitum. According to Wang et al. [17], each chick in every group was inoculated subcutaneously with 0.2 mL of *APEC* isolate suspension (1 × 10^8^ CFU/mL), calculated according to the McFerland standard [53]. Deaths and clinical signs of illness were recorded four times daily for 7 days post-inoculation (PI). The surviving chicks were killed at 7th day PI and the lesions were recorded. *APEC* isolates were classified, on the basis of their virulence degree for one day-old chicks, as follows: (a) highly pathogenic isolates—produced mortality or severe lesions including pericarditis, perihepatitis, air sacculitis, and liver necrosis in more than 50% of the challenged chicks, (b) intermediate pathogens—were nonlethal and produced lesions in fewer than 50% of the inoculates, and (c) low pathogens—produced no mortality and only occasional lesions in the air sacs [20].
- **Specific pathogen-free (SPF) embryonated chicken eggs (ECEs) model:** Fifteen groups of SPF ECEs (*n* = 10 eggs) for the same 13 isolates and 2 additional control groups (one group inoculated with saline and the other non-inoculated) were used in an embryo lethality test. According to Wooly et al. and Nolan et al. [13,21], 0.2 mL containing 500 CFU of each isolate (calculated according to McFerland standard [53]) was inoculated into 10 SPF ECEs through the allantoic sac (10-day old embryos); thereafter, by daily candling for 7 days PI, the mortality was recorded to classify *APEC* strains as follows: highly virulent strain resulted in mortality rate of >29% of ECEs, moderately virulent strains reported 10%–29% mortality, and <10% mortality was observed in a virulent strain.

### 4.5. In Vitro Antibiotic Sensitivity Testing of E. coli Isolates 

Fifteen molecularly confirmed *APEC* isolates were tested by disc diffusion on Mueller–Hinton agar (MHA) for sensitivity to 13 different antibiotics (Oxoid Laboratories, Basingstoke, Hampshire, England, and Lot No. 2230562), according to Finegold and Martin [54]. The tested antibiotics (discs) included ampicillin (AMP/10 µg), amoxycillin–clavulanic acid (AMC/30 µg), cefotaxime (CTX/30 µg), colistin (CT/10 µg), tetracyclines (TE/30 µg), doxycycline (DO/30 µg), gentamicin (CN/10 µg), neomycin (N/30 µg), spiramycin (SP/100 µg), florfenicol (FFC/30 µg), ofloxacin (OFX/5 µg), enrofloxacin (ENR/5 µg), and ciprofloxacin (CIP/5 µg). According to zone diameter interpretive standards for *Enterobacteriaceae* [22], inhibition zone diameters were measured and were evaluated. Zone diameters of colistin were interpreted according to what was suggested by [23]: {resistant ≤ 11 mm, sensitive ≥ 14 mm, as well as 12−13 mm, were considered susceptible}.

## 5. Conclusions

All tested *APEC* isolates (n = 13) in the northern delta of Egypt exhibited resistance to ampicillin, amoxycillin–clavulanic acid, and tetracyclines; moreover, two isolates (O55 and O158) showed resistance to all tested antibiotics. Of the isolates, 53.3% showed sensitivity to ofloxacin and enrofloxacin. Nine *E. coli* serotypes (O115, O142, O158, O55, O125, O114, O27, O20, and O15) were the most circulated ones. The molecular diagnosis of *APEC* is faster and more accurate than the traditional microbiological methods. In this study, it was difficult to correlate between studied virulence genes (*iss*, *fimH,* and *iutA)* and/or serotyping with in vivo virulence assessment models. Hence, further studies are warranted to investigate a wider range of virulence genes for their possible use in judging the virulence of *APEC*.

## Figures and Tables

**Table 1:** Different patterns of serotypes and virulence genes, and in vivo virulence assessment of *APEC* isolates.

Serial No.	Isolate Code No.	Serotype	Virulence Gene Content	In Vivo Virulence Assays	
				SPF 1-day-old Chicks ^a^	SPF ECEs ^b^
1	9	O115	*iss*	L	H
2	16	O115	*iss + iutA*	L	H
3	41	O158	*iss + fimH*	M	H
4	50	O158	*iss + fimH + iutA*	L	H
5	28	O114	*iss + iutA*	M	H
6	54	O114	*iutA*	L	H
7	5	O125	*iss*	H	H
8	2	O27	*iss*	M	H
9	7	O55	*iss + fimH*	L	H
10	39	O55	*iss + iutA*	H	H
11	15	O20	*iss + iutA*	L	H
12	32	O142	*iss + iutA*	L	H
13	49	O15	*iss*	L	H
14	Control ^c^	-	-	-	-
15	Control negative ^d^	-	-	-	-

^a^ SPF 1-day-old chicks (n = 5) were inoculated with each sample subcutaneously and were observed for 7 days post-infection to record the mortality rate. H: highly virulent (highly pathogenic isolates produced mortality or severe lesions including pericarditis, perihepatitis, air sacculitis, and liver necrosis in more than 50% of the challenged chicks), M: moderately virulent (were nonlethal and produced lesions in fewer than 50% of the inoculates), L: low virulent (produced no mortality and only occasional lesions in the air sacs) [20]. ^b^ 10-day-old SPF ECEs (n = 10) were inoculated with each sample via the allantoic sac route and were observed for 6 days post-inoculation to record the mortality rate. H: highly virulent strain resulted in mortality rate of >29% of ECEs; M: moderately virulent strains reported 10–29% mortality, and L: low virulent strains induced <10% mortality [13,21]. ^c^ Control for each assay (1-day-old chicks or ECEs) were sham inoculated with saline by the same route and dose; neither mortality nor post-mortem lesions were observed. ^d^ Control negative for each assay (1-day-old chicks or ECEs were not inoculated).

**Table 2:** Results of antibiotic sensitivity testing for *APEC* isolates.

Antibiotic ^a^	*APEC* Isolates (Code No.)													*% of Isolates* ^b^		
	9	16	41	50	28	54	5	2	7	39	15	32	49	R	I	S
Ampicillin	R	R	R	R	R	R	R	R	R	R	R	R	R	100	0	0
Amoxycillin–clavulanic acid	R	R	R	R	R	R	R	R	R	R	R	R	R	100	0	0
Cefotaxime	R	I	I	R	R	R	R	R	I	R	S	R	I	61.54	30.77	7.69
Colistin	R	R	R	R	R	R	R	R	R	R	S	R	R	92.31	*	7.69
Tetracyclines	R	R	R	R	R	R	R	R	R	R	R	R	R	100	0	0
Doxycycline	S	R	R	R	R	R	R	I	R	R	R	R	R	84.62	7.69	7.69
Gentamicin	R	S	R	R	S	S	S	R	R	S	S	R	R	53.85	0	46.15
Neomycin	R	I	R	I	S	R	I	I	R	I	I	R	I	38.46	53.85	7.69
Spiramycin	R	R	I	R	R	R	I	R	R	R	R	R	R	84.62	15.38	0
Florfenicol	R	I	R	R	R	R	S	S	R	S	R	R	R	69.23	7.69	23.7
Ofloxacin	I	S	S	R	I	S	R	R	R	S	S	S	S	30.77	15.38	53.85
Enrofloxacin	R	S	S	R	R	S	R	R	R	S	S	S	S	46.15	0	53.85
Ciprofloxacin	R	I	S	R	R	I	R	R	R	S	S	I	R	53.84	23.08	23.08

^a^ Oxoid Laboratories, Basingstoke, Hampshire, England, and Lot No. 2230562. ^b^ R: resistant, I: intermediate sensitive, and S: sensitive to antibiotics, according to [22]. * Zone diameters for colistin were interpreted according to what was suggested by [23] as {R ≤ 11 mm, S ≥14 mm, as well as 12–13 mm, were considered susceptible}.

**Table 3:** The history of investigated broiler chick flocks.

Code No.	Location	Total Bird No.	Age (days)	Breed	PM Lesions	Antibiotics during Last 3 days
**1**	Beheira	2000	14	Balady	Air sacculitis, unabsorbed yolk sac, and precipitation of urates on ureters	Florfenicol
**2**	Beheira	4000	12	Arbo Acres	Air sacculitis and fibrinous pericarditis and perihepatitis	Tylosin and colistin
**3**	Beheira	6000	11	Ross	Air sacculitis, pneumonia, fibrinous pericarditis, and perihepatitis and unabsorbed yolk sac	Tylosin and florfenicol
**4**	Beheira	6000	9	Balady	Unabsorbed yolk sac	Ciprofloxacin and florfenicol
**5**	Beheira	5000	9	Arbo Acres	Air sacculitis and whitish diarrhea	Sulphadiazine sodium plus trimethoprim and ciprofloxacin
**6**	Beheira	26,000	6	Cobb	Air sacculitis and unabosorbed yolk sac	Tiamulin and colistin
**7**	Beheira	6000	12	Ross	Air sacculitis and fibrinous pericarditis and perihepatitis, unabsorbed yolk sac	Ciprofloxacin and colistin
**8**	Beheira	6000	14	Cobb	Caseated blug at tracheal bifurcation and pneumonia	Florfenicol
**9**	Beheira	2500	12	Cobb	Ricketts and unabsorbed yolk sac	Florfenicol
**10**	Beheira	100	4	Cobb	Air sacculitis, fibrinous pericarditis and perihepatitis, unabsorbed yolk sac	Colistin
**11**	Beheira	9000	14	Cobb	Air sacculitis, septicemia, and unabsorbed	Tilmicosin and colistin
**12**	Beheira	1000	10	Ross	Unabsorbed yolk sac, air sacculitis	Tilmicosin and colistin
**13**	Beheira	1000	7	Cobb	Air sacculitis and pneumonia	Cefotaxim injection
**14**	Beheira	6000	6	Ross	Omphalitis, air sacculitis	Florfenicol
**15**	Beheira	100	7	Cobb	Pneumonia, air sacculitis, and omphalitis	Colistin
**16**	Beheira	10,000	12	Cobb	Enteritis and air sacculitis	Tylosin and colistin
**17**	Beheira	450	11	Cobb	Enteritis and air sacculitis	Colistin and doxy
**18**	Beheira	1000	15	Hybrid	Precipitation of urates on ureters	Sulphadiazine sodium plus trimethoprim
**19**	Beheira	5000	9	Ross	Air sacculitis, pneumonia, fibrinous pericarditis and perihepatitis and precipitation of urates on ureters	Sulphadiazine sodium plus trimethoprim
**20**	Beheira	4000	7	Ross	Air sacculitis and enteritis	Sulphadiazine sodium plus trimethoprim
**21**	Beheira		10	Cobb	Enteritis	Sulphadiazine sodium plus trimethoprim
**22**	Beheira	2700	15	Ross	Pneumonia and airsacculitis	Florfenicol
**23**	Beheira	3400	14	Ross	Air sacculitis, fibrinous pericarditis and perihepatitis	Florfenicol
**24**	Beheira	2000	15	Cobb	Enteritis	Colistin and doxycycline
**25**	Beheira	1500	14	Ross	Enteritis	Colistin and tylosin
**26**	Beheira	1000	13	Cobb	Enteritis	Colistin and doxycycline
**27**	Beheira	4500	15	Cobb	Mycotoxicosis, air sacculitis, fibrinous pericarditis, and perihepatitis	Sulphadiazine sodium plus trimethoprim and cefotaxime
**28**	Alexanderia	9000	15	Cobb	Lesions of Gumboro disease and enteritis	Florfenicol
**29**	Beheira	10,000	5	Ross	Enteritis	Florfenicol and tylosin
**30**	Alexanderia	8000	15	Cobb	Necrotic enteritis	Florfenicol
**31**	Beheira	7000	9	Ross	Enteritis	Florfenicol
**32**	Beheira	10,000	10	Cobb	Enteritis	Tylosin and colistin
**33**	Beheira	10,000	15	Ross	Lesions of NewCastle and omphalitis	Doxycycline and colistin
**34**	Beheira	1000	15	Cobb	Enteritis	Doxycycline and colistin
**35**	Beheira	1000	14	Cobb	Air sacculitis	Florfenicol and doxycycline
**36**	Beheira	2700	15	Ross	NewCastle and Gumboro diseases IBD	Florfenicol
**37**	Beheira	1000	6	Cobb	Omphalitis, airsacculitis	Colistin and tylosin
**38**	Beheira	6000	15	Cobb	Air sacculitis, fibrinous pericarditis and perihepatitis	Florfenicol
**39**	Beheira	5000	6	Arbo Acres	Air sacculitis	Florfenicol and doxycycline
**40**	Beheira	5000	6	Arbo Acres	Air sacculitis	Ciprofloxacin and florfenicol
**41**	Beheira	4000	3	Ross	Omphalitis	Doxycycline and colistin
**42**	Beheira	2000	4	Ross	Omphalitis	Florfenicol
**43**	Beheira	600	13	Arbo Acres	Pneumonia, air sacculitis, fibrinous pericarditis and perihepatitis	Florfenicol
**44**	Beheira	4000	4	Ross	Enteritis	Tylosin and colistin
**45**	Beheira	8500	14	Cobb	Airsacculitis	Doxycycline and colistin
**46**	Beheira		10	Arbo Acres	Air sacculitis, fibrinous pericarditis, perihepatitisand omphalitis	Ciprofloxacin and florfenicol
**47**	Beheira	9000	2	Arbo Acres	Omphalitis	Florfenicol and doxycycline
**48**	Beheira	5000	3	Cobb	Pneumonia, air sacculitis, fibrinous pericarditis, and perihepatitis	Tylosin and colistin
**49**	Beheira	3000	6	Cobb	Air sacculitis, fibrinous pericarditis, perihepatitisand unabsorbed yolk sac	Tylosin and colistin
**50**	Beheira	5000	10	Cobb	Air sacculitis and omphalitis	Doxycycline and colistin
**51**	Beheira	1000	15	Arbo Acres	Necrotic enteritis and air sacculitis	Ciprofloxacin and florfenicol
**52**	Beheira	10,000	10	Cobb	Air sacculitis	Doxycycline and colistin
**53**	Beheira	1000	15	Cobb	Air sacculitis	Florfenicol
**54**	Beheira	9000	15	Arbo Acres	Enteritis and slight airsacculitis	Doxycycline and colistin

**Table 4:** Group O somatic antisera for serological identification of *E. coli* isolates.

Polyvalent Sera	Monovalent Sera						
**Polyvalent 1**	O1	O26	O86	O111	O119	O127	O128
**Polyvalent 2**	O44	O55	O125	O126	O146	O166	
**Polyvalent 3**	O18	O114	O142	O151	O157	O158	
**Polyvalent 4**	O6	O27	O78	O148	O159	O168	
**Polyvalent 5**	O20	O25	O63	O153	O167		
**Polyvalent 6**	O8	O15	O115	O169			
**Polyvalent 7**	O28	O112	O124	O136	O144		
**Polyvalent 8**	O29	O143	O152	O164			

**Table 5:** Oligonucleotide primer sequences.

Gene		Primer Sequence (5′-3′)	Amplified Product (bp)	Reference
**Species-specific**	***phoA***	CGATTCTGGAAATGGCAAAAG CGTGATCAGCGGTGACTATGAC	720 bp	[49]
**Virulence**	***iss***	ATG TTA TTT TCT GCC GCT CTG CTA TTG TGA GCA ATA TAC CC	266 bp	[50]
	***iutA***	ATGAGCATATCTCCGGACG CAGGTCGAAGAACATCTGG	587 bp	[51]
	***fimH***	TGCAGAACGGATAAGCCGTGG GCAGTCACCTGCCCTCCGGTA	508 bp	[52]

**Table 6:** Cycling conditions for PCR

Gene		Initial Denaturation	Denaturation	Annealing	Extension	Number of Cycles	Final Extension
**Species-specific**	***phoA***	94 °C 5 min	94 °C 30 s	58 °C 45 s	72 °C 45 s	35	72 °C 10 min
**Virulence**	***Iss***	94 °C 5 min	94 °C 30 s	58 °C 45 s	72 °C 45 s	35	72 °C 10 min
	***fimH***	95 °C 2 min	94 °C 30 s	58 °C 30 s	72 °C 1 min	33	72 °C 7 min
	***iutA***	94 °C 3 min	94 °C 1 min	55 °C 1 min	72 °C 30 s	30	72 °C 7 min
